# Chikungunya Viral Fitness Measures within the Vector and Subsequent Transmission Potential

**DOI:** 10.1371/journal.pone.0110538

**Published:** 2014-10-13

**Authors:** Rebecca C. Christofferson, Daniel M. Chisenhall, Helen J. Wearing, Christopher N. Mores

**Affiliations:** 1 Department of Pathobiological Sciences, School of Veterinary Medicine, Louisiana State University, Baton Rouge, Louisiana, United States of America; 2 Department of Biology, The University of New Mexico, Albuquerque, New Mexico, United States of America; 3 Department of Mathematics & Statistics, The University of New Mexico, Albuquerque, New Mexico, United States of America; Singapore Immunology Network, Agency for Science, Technology and Research (A*STAR), Singapore

## Abstract

Given the recent emergence of chikungunya in the Americas, the accuracy of forecasting and prediction of chikungunya transmission potential in the U.S. requires urgent assessment. The La Reunion-associated sub-lineage of chikungunya (with a valine substitution in the envelope protein) was shown to increase viral fitness in the secondary vector, *Ae. albopictus*. Subsequently, a majority of experimental and modeling efforts focused on this combination of a sub-lineage of the East-Central-South African genotype (ECSA-V) – *Ae. albopictus*, despite the Asian genotype being the etiologic agent of recent chikungunya outbreaks world-wide. We explore a collection of data to investigate relative transmission efficiencies of the three major genotypes/sub-lineages of chikungunya and found difference in the extrinsic incubation periods to be largely overstated. However, there is strong evidence supporting the role of *Ae. albopictus* in the expansion of chikungunya that our R0 calculations cannot attribute to fitness increases in one vector over another. This suggests other ecological factors associated with the *Ae. albopictus-*ECSA-V cycle may drive transmission intensity differences. With the apparent bias in literature, however, we are less prepared to evaluate transmission where *Ae. aegypti* plays a significant role. Holistic investigations of CHIKV transmission cycle(s) will allow for more complete assessment of transmission risk in areas affected by either or both competent vectors.

## Introduction

Public health threats from arboviruses have become increasingly problematic for the United States in the last decade. The summer of 2012 saw high transmission intensity of West Nile Virus (WNV), most notably in Dallas, TX [1]. Dengue virus (DENV) has been repeatedly introduced into South Texas, South Florida, and New York [2]–[5]. And while these DENV introductions may result in limited transmission, South Florida has significant transmission events in 2009 when DENV-1 established in Key West [6], [7]. In 2013, a genetically distinct DENV-1 was introduced separately into Martin County and resulted in sustained transmission over the summer months [8].

Even more recent is the emergence of chikungunya (CHIKV) in the Americas. First detected in St. Martin, it quickly spread throughout the Caribbean [7]. The number of cases rapidly grew to the tens of thousands over the course of a handful of months and numerous imported cases have been detected in the U.S. [9]. CHIKV is a relatively recent threat. First identified in Tanzania in the 1950s [10], it has not been as extensively studied as DENV, which shares a similar *Ae. aegypti-*driven transmission cycle. As such, the accuracy of forecasting and prediction of CHIKV emergence in the Americas requires urgent assessment.

After the outbreak of CHIKV on La Reunion Island in 2006 [11], a variant of the East-Central-South-African (ECSA) genotype was identified with an amino acid substitution on the envelope (E1) at position 226 (alanine to valine, sub-lineages ECSA-A and ECSA-V, respectively) and this mutation was shown to increase the virus’ fitness in a historically secondary vector, *Ae. albopictus* [12], [13]. This increase in efficiency manifested in a decrease in the extrinsic incubation period (EIP, the time it takes for a mosquito to become infectious after exposure via viremic bloodmeal) in this mosquito species, and was used to explain in part the apparent dominance of the ECSA genotype over the Asian genotype in subsequent outbreaks [14]–[17]. In some of these same epidemics, the E226A sub-lineage persisted in the areas where *Ae. aegypti* is predominant, indicating that the E226 V-*albopictus* combination could not completely displace the E226A-*aegypti* transmission pairing [15].

However, after this shift in transmission ecology and distribution, a large proportion of experimental and modeling efforts focused on the combination of ECSA-V in *Ae. albopictus* (details in Metadata Results below), despite the Asian genotype being the etiologic agent of recent CHIKV outbreaks in China, the Philippines, Indonesia and the Caribbean [7], [18], [19].

As there is no vaccine available for CHIKV, the focus of intervention efforts will necessarily be on prevention of introductions and emergence in the United States through vector control and avoidance campaigns. An influential component of forecasting or predictive capabilities is our understanding of the efficiency of CHIKV in the two vector species present in the United States, *Ae. aegypti* and *Ae. albopictus* without bias. We hypothesized that available, published data did not support the apparent assumption that an increase in fitness of ECSA-V in *Ae. albopictus* has been the driving factor of transmission differences. Thus, we explore here a collection of data comparing the relative efficiency of the two major genotypes (and sub-lineages of ECSA) of CHIKV in these mosquito species to determine whether there is a meaningful difference among these genetic variants. Detecting any such differences (or lack thereof) will better inform modeling capabilities (heavily biased towards ECSA-V:*albopictus*), and identify gaps in our CHIKV knowledge base, indicating avenues of future investigation.

## Materials and Methods

We utilized Google Scholar and PubMed to search combinations of the following terms: chikungunya, models, transmission, prediction, and mathematical models, which returned 14 articles that directly consider the efficiency of the virus in the vector by explicitly modeling the extrinsic incubation period in CHIKV transmission [20]–[33]. Of these, 71.4% (10/14) focused exclusively on transmission based on the combination of *Ae. albopictus* and the ECSA-V sub-lineage either directly by informing parameters from experimental data, indirectly by parameterizing models based on the La Reunion outbreak of 2005-6, or both [20]–[26], [29]–[32]. Only one paper looked at CHIKV dynamics in both mosquito species [22] and two papers modeled CHIKV transmission dynamics in *Ae. aegypti* only [27], [28]. This again highlights the bias towards the La Reunion outbreak dynamics, which is understandable as it was this outbreak that generated the renewed interest in CHIKV.

Considering the bias in the model literature, we next conducted a search on Google Scholar and PubMed using various combinations of the following search terms: chikungunya, vector competence, *Aedes aegypti, Aedes albopictus,* extrinsic incubation period. The search returned over 130 articles. Some papers did not present data in absolute numeric (as opposed to only ratios), did not identify the day post exposure on which dissemination was evaluated, while others were not included because they utilized recombinant or otherwise genetically modified virus or hybrid mosquitoes, investigated co-infections within the mosquito (viral or parasitic), subjected mosquitoes to extreme temperatures, or investigated other routes of transmission (i.e. venereal). In addition, our exploration was restricted to the two genotypes of CHIKV responsible for the majority of outbreaks: the ECSA and the Asian genotypes for a total of 22 studies included [12], [13], [34]–[53]. These studies infected mosquitoes orally with virus titer ranging from 10^4.3–8^ pfu/ml and incubation temperatures between 26 and 30C. Data was censored for those studies that fed titers between 10^5.5^ and 10^7.3^, but this did not affect distribution fits and thus all 22 studies were included here.

### Comparison of viral efficiency in two vector species

Our model framework has been previously published [54] and we used this framework to calculate the basic reproductive number (R0) for each of the scenarios (1). Human infectiousness (viremia curve) and acquisition curves (representing the probability that a mosquito imbibes virus in the bloodmeal) were constructed using data from [55], [56] as in [54]. It is important to note that acquisition does not take into account phenomena that alter the down-stream process of vector competence (e.g., midgut barrier, salivary gland barrier, viral efficiency differences). It more accurately represents vertebrate competence, which we have chosen to hold constant here to focus on these potential viral efficiency differences in the vector. There is no study where mosquitoes have been allowed to feed on CHIK-infected, viremic individuals. Thus, we are assuming that the rate of acquisition is viral type-blind, based on the number of viral particles circulating in the bloodstream, and therefore not significantly different among arboviruses. While this is potentially a large assumption, altering the value of q (should more data provide specific estimates) would result in proportional changes to the R0 values for these combinations of mosquitoes and viral strains. Thus, the interpretation of differences would remain the same. We base our acquisition:viremia rate on DENV-1 from [56]. All calculations and curve fitting were performed in R version 3.0.1 (stats package).

The R0 formulation is given by:
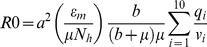
where **m = 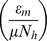
** is the mosquito density (mosquitoes/person), **a** is the biting rate (number of times a female will bite/day), **b** ^−1^ is the average EIP of the virus in the mosquito (b is the rate parameter of the cumulative exponential distribution), **µ** ^−1^ is the average lifespan of the mosquito, **q_i_** is the acquisition of virus by the mosquito (dependent on viremia over interval i), and **v_i_** is the length of interval *i* in days.

Model parameters are given in Table 1. To compare viral efficiency in the two species directly, all parameters were held constant except for b^−1^ and µ^−1^, which varied according to species of mosquito and genotype and sub-lineages of CHIKV, based on the metadata.

**Table 1:** Values, definitions and sources for parameters used in modeling efforts.

Parameter (value)	Definition	References
a (.38)	Biting rate	Average for both spp. and held constant [22]
b^−1^ (variable)	Average extrinsicincubation period	Calculated from metadata
µ^−1^ (19.5 days)	Average mosquitolifespanfor *Ae. aegypti* and *Ae.* *albopictus*,	Average for both spp. and held constant [22]
m (1.9mosquitoes/human)	Mosquito density	[58]
v_1–8_ (1 day each)	Duration of eachInfectioussubclass (1–8)	[55]
q_1_ (9.07e-01)	Acquisition potentialrelative to viremia	Viremia from [55] and associated acquisition potential calculated from [56]
q_2_ (8.52e-01)		
q_3_ (2.24e-01)		
q_4_ (6.20e-02)		
q_5_ (1.13e-02)		
q_6_ (4.48e-03)		
q_7_ (5.73e-03)		
q_8_ (4.81e-03)		

Data points were divided by mosquito species (*Ae. aegypti* versus *Ae. albopictus*) and genotype of CHIKV (Asian versus ECSA). The ECSA genotype was further subdivided based on the mutation at the E226 position. Those strains that did not have the amino acid shift were coded as ECSA-A and those with the La Reunion amino acid shift were coded as ECSA-V.

We fit cumulative exponential distributions to the compiled meta-data by first averaging all points per day post exposure for each lineage-mosquito combination. Default parameterization of vector competence and EIP in models assumes the exponentially distributed EIP, which requires a single rate parameter [20]–[27], [29]–[33]. This rate parameter is the inverse of the average EIP 
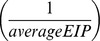
. We estimated specific rates (using the fitdist package and moment matching estimation) for each mosquito-genotype (and sub-lineages where applicable) combination and determined the average rate of transition from the exposed to the infectious class. We then employed a bootstrapping re-sampling (n = 5000 iterations) technique to obtain the 95% confidence intervals of these rates and transformed them to average EIP (in days) and the associated 95% CI.

## Results

### Metadata exploration reveals bias in literature (Figures S1–S3)

**Figure 1:**
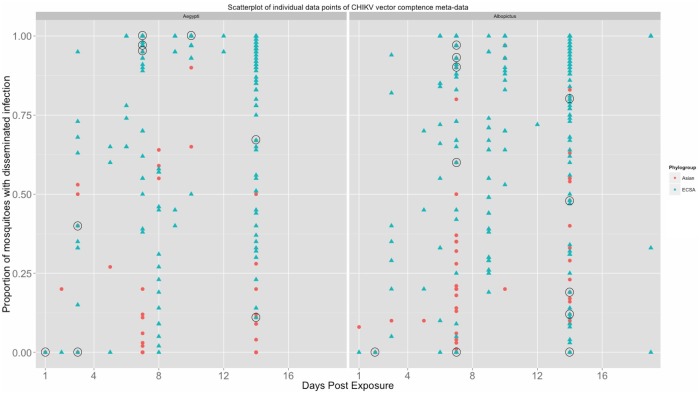
Scatterplot depicting the data points from mosquito infection experiments the Asian (red dots) versus ECSA (green triangles) genotype where circles indicate an overlap where both Asian and ECSA data point exists.

**Figure 2:**
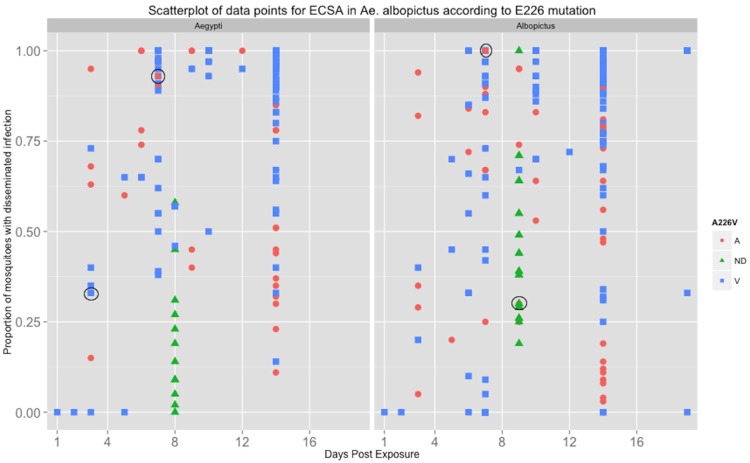
Scatterplot depicting the data points from mosquito infection experiments ECSA-A (red dots) versus ECSA-V (blue squares) with those data of non-determined lineage (green triangles). Circles indicate where there is an overlap of at least two sub-lineages.

Of the vector competence studies, 22.7% looked at *Ae. albopictus* only, 18.2% looked at *Ae. aegypti* only, and 59.1% looked at both mosquito species (Figure S1). With regard to CHIKV genotype, 81.8% of studies investigated the ECSA genotype only while only 1 study looked at the Asian genotype only; however, 13.6% (n = 3) studies studied both genotypes (Figure S2). Sixteen studies (72.7%) investigated ECSA in *Ae. aegypti* (9/16 ECSA-V only, 2/16 ECSA-A only, 1 not determined, and 4/16 considered both ECSA-A and ECSA-V). Sixteen studies also delineated between ECSA-A and V in *Ae. albopictus.* Of these 16, 62.5% (10/16) investigated ECSA-V only (all since 2008), 25% considered both sub-lineages and 1 study looked at the ECSA-A sub-lineage only (with 1 study not determined) (Figure S3). Further demonstration the data bias towards the *Ae. albopictus* ECSA-V combination is displayed in the scatter plot (Figures 1 and 2) of all data points from the 22 papers. Figure 1 shows that the ECSA genotype in general has been more studied than the Asian genotype and Figure 2 shows that within the ECSA genotype, the ECSA-V sub-lineage has received the most attention in both mosquito species.

Also apparent in these figures is the lack of any clear delineation in viral fitness among the genotype/sub-lineages of CHIKV. The scatter along the y-axis (proportion of tested mosquitoes reported with a disseminated infection) does not support the supposition of a significant increase in fitness of the ECSA-V sub-lineage in *Ae. albopictus*, nor does it indicate there is a clear difference among any combination of genotype/sub-lineage and mosquito vector species.

**Figure 3:**
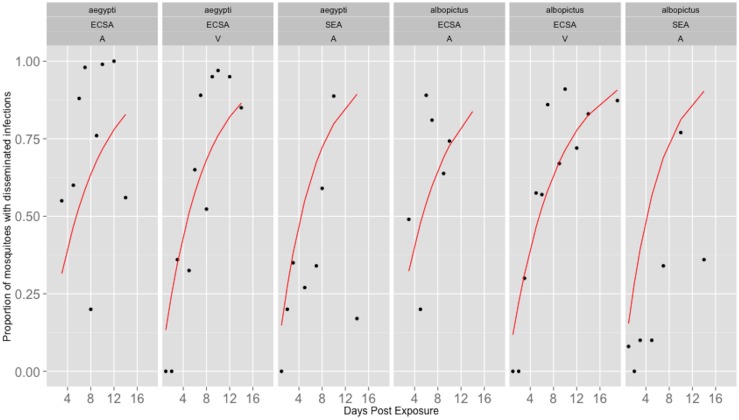
The average proportion of mosquitoes with disseminated infections from all 23 studies (black dots) were used to fit the average rate of dissemination, estimated by the cumulative exponential distribution (red line).

The daily average vector competence from the 22 studies was plotted against to the corresponding day post exposure (Figure 3). The estimated rates of movement from the exposed to infectious classes based on these fits and the confidence intervals from the bootstrap method are given in Table 2. In addition, the back-calculated average EIP and associated confidence intervals are also given in Table 2. These calculated average EIP values range between 5.9 and 8.2 days. Further, the EIP differences do not translate to large differences in epidemic potential, as measured by R0, which range from 7.81–8.49. These results indicate that differences in transmission potential are not significantly affected by the small differences in EIP and are given in Table 3.

**Table 2:** Estimates of the average rate of transition from Exposed class to Infectious class and corresponding EIP of each (sub) lineage:mosquito combination.

genotype	Mosquito	Rate (b) (95% CI)	Avg. EIP (b^−1^) in days (95% CI)*
Asian	*Ae. aegypti*	0.160 (0.088, 0.364)	6.25 (2.75, 11.36)
ECSA-A		0.122 (0.069, 0.261)	8.20 (3.83, 14.50)
ECSA-V		0.143 (0.086, 0.295)	6.99 (3.39, 11.63)
Asian	*Ae. albopictus*	0.167 (0.098, 0.415)	5.99 (2.41, 10.20)
ECSA-A		0.130 (0.069, 0.322)	7.69 (3.11, 14.50)
ECSA-V		0.125 (0.075, 0.246)	8.00 (4.07, 13.10)

*As rate and EIP are inverses, the confidence intervals are also inverses. That is, the lower CI limit for the rate value is the upper CI limit for the EIP value.

**Table 3:** R0 values calculated based on differences in viral efficiency among (sub) lineage:mosquito combinations.

Lineage:mosquito Combination	R0
Asian:*aegypti*	8.39
ECSA-A:*aegypti*	7.81
ECSA-V:*aegypti*	8.17
Asian:*albopictus*	8.49
ECSA-A:*albopictus*	7.96
ECSA-V:*albopictus*	7.87

## Discussion

Published studies of CHIKV vector competence and EIP are skewed towards investigations of the ECSA genotype, which is not surprising given that 81.8% (20/23) of studies were published after the large outbreak on La Reunion [11], where that genotype of CHIKV was purported to have adapted to *Ae. albopictus* through the A226 V envelope mutation. The bias in subsequent literature is clear, and the assumption has been that *Ae. albopictus* and the ECSA-V sub-lineage were of primary concern because of the large scale of the La Reunion outbreak and the subsequent spread of the ECSA-V sub-lineage throughout the Indian Ocean and into Southeast Asia [57]. However, in December 2013, it was not this ECSA genotype, but a strain of the Asian genotype that was introduced into the Caribbean and quickly established [7]. For this reason, it is likely that the Asian genotype that poses the most immediate threat to the mainland United States. However, only 4 out of the 22 studies investigated the Asian lineage in either mosquito [35], [48], [50], [53]. Given the apparent insignificant difference in vector efficiency between *Ae. aegypti* and *Ae. albopictus* and the widespread distribution of *Ae. albopictus* in the U.S., the bias in data is potentially misleading.

As vector competence has become increasingly recognized as a dynamic, temporal process [58]–[61], we were interested to determine how many of the experimental studies took this approach when evaluating CHIKV vector competence and EIP. Of the 22 studies, 59.1% (13/22) of the studies assessed vector comp/EIP at a single time point, 18.2% (4/22) had 2 time points, and 22.7% (5/22) had 3 or more time points (Figure S4). If time post exposure was defined as “early” (a week or less) or “late” (anything more than 7 days post exposure), 9.1% (2/22) looked at early only, 77.3% (17/22) looked at late only, and 13.6% looked at both stages (3/22) (Figure S5). Multiple time points are critical when investigating the average rate of transition from exposed to infectious mosquitoes, used to predict and forecast transmission dynamics and potentially inform policy. We also recognize that vector competence varies among populations of mosquitoes of the same species, as demonstrated in [53]. However, we found no discernable pattern when we explored the data delineating on geographic (or ‘colony’, if applicable) origin of the mosquito used in each study (Figure S6). Likely the geographical differences in mosquito competence would be more useful for very spatially detailed model efforts, which our R0 calculations are not. If appropriate, more detailed hypotheses and analyses should, however, make effort to explore this phenomenon.

When we fit the available data as such (assuming an exponentially distributed EIP), we found no meaningful differences in the rates of mosquito transition from exposed to infectious among the genotype/lineage – mosquito species combinations. The ECSA-V:*albopictus* combination EIP averaged 8 days, which is one of the higher values. This is especially important as most modeling studies focus on this combination and assume a 1–3 day extrinsic incubation period within the mosquito, when in fact this experimental data represents the earliest dissemination point, not the average [21]–[26], [62]. This suggests that the epidemic potential resulting from direct calculations of R0 and the epidemic dynamics from these models may be overestimated as much as 26% (R0_[1,3] day EIP_ = [10.54,9.6]). This would affect not only the estimations of emergence potential, but would likely also alter the estimation of the epidemic intensity and timing. Together, errors in these metrics would affect policy decisions such as resource allocation and timing of vector control measures.

However, the success of the ECSA-V:*albopictus* combination has been well documented [15]–[17], [63]–[65]. Recently, a study explored the difference in the indirect biting rates of the two vector species, *Ae. aegypti* and *Ae. albopictus* [66]. Unsurprisingly, the study found that in urban areas, *Ae. aegypti* landed on the collector almost 4 times more than *Ae. albopictus,* while in a suburban area there were no *Ae. aegypti* and the indirect biting rate of *Ae. albopictus* was over 15/human/day [66]. Many of the outbreaks of CHIKV have implicated the ECSA-V lineage and *Ae. albopictus* as the primary virus and vector, respectively. However, examination of the human population affected shows that in many of these outbreaks, the focus of transmission was in suburban areas or peri-rural areas such as rubber plantations [15], [16], [64]. Further, *Ae. albopictus* has been implicated as the primary vector in places where either it has an ecological-associated advantage over *Ae. aegypti* [63] or is the only competent *Aedes* species present, such as in more temperate climates [17]. Thus, it is not necessarily the viral dynamics in *albopictus* that have driven the CHIKV outbreaks so much as the interactions of the human and mosquito ecologies. For example, the mortality rates of the two mosquitoes species is purported to be different, as well as the biting rates [22], and although there are no consistent estimates of the densities of c0oincident populations of the two *Aedes* species, studies have shown that there is a large difference in the density of each species relative to the human population, often relying on larval counts or other indirect methods (i.e. landing rates). For *Ae. aegypti*, a mosquito density was reported and averaged to be 2.23/person (censoring extreme observations) from [67], and the density for *Ae. albopictus* ranged from.85 to 80/person [68]–[70]. All of these factors may influence the enhancing role of *Ae. albopictus* in CHIKV transmission, perhaps more so than the efficiency differences.

Of particular interest is the mortality rate of the mosquito, as it is critical to evaluating fitness differences [58], [61], [71], [72]. Here, we assumed an average lifespan of 19.5 days in our R0 calculations, which means that the ratio of lifespan to EIP is relatively large here. Thus, we briefly investigated the role of lifespan in the context of the differences in EIP, and the affect on corresponding disparities in R0. Since only the rate parameter *b* and µ would change in our calculations (we hold all other R0 parameter constant), we can look at the proportional change in 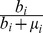 to determine how mortality rates µ^−1^ and these transition rates (EIP) from exposed to infectious classes modulate the differences in R0.
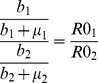



For example, the difference in EIP in the Asian:*aegypti* combination (EIP = 6.25 days) to the ECSA-V:*albopictus* combination (EIP = 8.00 days) corresponds to an approximate 7% greater R0 value for the Asian:a*egypti* combination. As the mortality rate of the mosquito is decreased (thus the ratio of lifespan to EIP gets closer to 1), the advantage of the Asian:*aegypti* combination also increases (comparatively 13% greater R0 value when µ^−1^ = 7.5 days). Indeed, for the R0 difference between the Asian:*aegypti* and ECSA-V:*albopictus* combinations to be less than 5%, the average lifespan of the mosquito(es) would need to be greater than 28.5 days. So while mosquito mortality is paramount to assessing differences in EIP and viral efficiency within the mosquito, likely the other ecological factors are more critical drivers of the transmission intensity attributed to the ECSA-V:*albopictus* combination.

## Conclusions

The 2006 outbreak on La Reunion Island likely was the impetus for renewed and expanded interest in CHIKV. Subsequent discovery of the E226 mutation (A→V) and the reported increased efficiency in *Ae. albopictus* [12] lead to an apparent bias in the literature, experimental and theoretical alike. This compilation of available experimental CHIKV vector competence data indicates that there is a similar efficiency among the genotypes and sub-lineages in both mosquito species. Thus, the species of mosquito and differences in behavior, habitat, etc. (as parameterized by contact rate with humans) may be the most critical defining factors of transmission intensity. However, there are several apparent differences in the data and transmission model outputs. This prompts several observations:

1. Genotype (i.e. ECSA, Asian) is likely too coarse a characterization on which to accurately assess transmission intensity, as is the case where closely related DENV strains have been shown to have different transmission results [5].
2. Most models implicitly assume exponentially distributed EIP, which should be parameterized with the average EIP, not the earliest detection times (as in the case of ECSA-V:*albopictus* EIP of 1–3 days) [22]–[25].
3. Relatedly, the cumulative exponential parameterization of EIP is not the best distributional assumption [28], [58], [73] for modeling the transition of mosquitoes from exposed to infectious, as it might be that differences on the fringes of this distribution may be most important.
4. More focus should be placed on the context of transmission differences (ecological, i.e.), similar to the One Health initiative, which places an emphasis on the interaction of the environment in zoonotic disease transmission.

There is very strong evidence supporting the role of *Ae. albopictus* in the expansion of CHIKV, especially into more temperate and/or suburban areas [14]–[17]. The clear data bias in the literature, however, does not sufficiently account for the entirety of CHIKV transmission ecology, because there are few experimental or modeling efforts that address the Asian genotype and other sub-lineages in the primary vector *Ae. aegypti*. This means that we are less prepared to evaluate transmission where *Ae. aegypti* still play a significant role or where this species is implicated as the primary transmission vector [7]. Indeed, as *Ae. aegypti* has been implicated in the dengue introduction in Key West, FL and in transmission along the US-Mexican border of Texas [3], this is an issue directly speaking to the public health security of the southern United States.

In summary, it is imperative that experiments account for the utility of time-course data when describing a dynamic process such as vector competence and EIP identification. Models, on the other hand, would benefit from recognizing the context of the biological data and carefully vet the assumptions of parameters developed from these data. Multi-disciplinary efforts and better cross-discipline understanding are key to more accurate forecasts, better informed policy decisions, and ultimately a less at-risk populace.

## Supporting Information

Figure S1 **The number of vector competence data points (y-axis) for each study (x-axis).** The papers are subdivided (colored bars) depending on whether the data was for *Ae. aegypti* or *Ae. albopictus* or both.
(TIFF)

Figure S2 **The number of vector competence data points (y-axis) for each study (x-axis).** Studies are divided (colored bars) depending on whether the data is for the Asian or ECSA genotype.
(TIFF)

Figure S3 **The number of vector competence data points (y-axis) for each study (x-axis).** The studies are divided (colored bars) based on whether the data corresponds to the ECSA-A (A), ECSA-V (V) sublineage of the ECSA genotype or if the dilineation in the ECSA genotype was not determined (ND).
(TIFF)

Figure S4 **The number of vector competence data points (y-axis) for each study (x-axis) divided (colored bars) by the day on which the data point was assessed.**
(TIFF)

Figure S5 **The number of vector competence data points (y-axis) for each study (x-axis) divided (colored bars) depending on whether the dissemination determination was done during the early stage of infection (≤7 days post exposure) or late stage (>7 days).**
(TIFF)

Figure S6 **Scatterplot of data points when subdivided by CHIKV genotype (columns) and mosquito species (rows).** Color denotes the continental or regional origin of the mosquito strains utilized (or denoted as ‘colony’ if applicable) and shape of the point denotes sub-lineage of ECSA (A or V) or ND (not-determined) if Asian genotype.
(TIFF)
