# Supplementary figures and images for: Chikungunya Viral Fitness Measures within the Vector and Subsequent Transmission Potential
Source: PLoS One. 2014 Oct 13;9(10):e110538. doi: 10.1371/journal.pone.0110538 (PMC4195746; doi:10.1371/journal.pone.0110538)

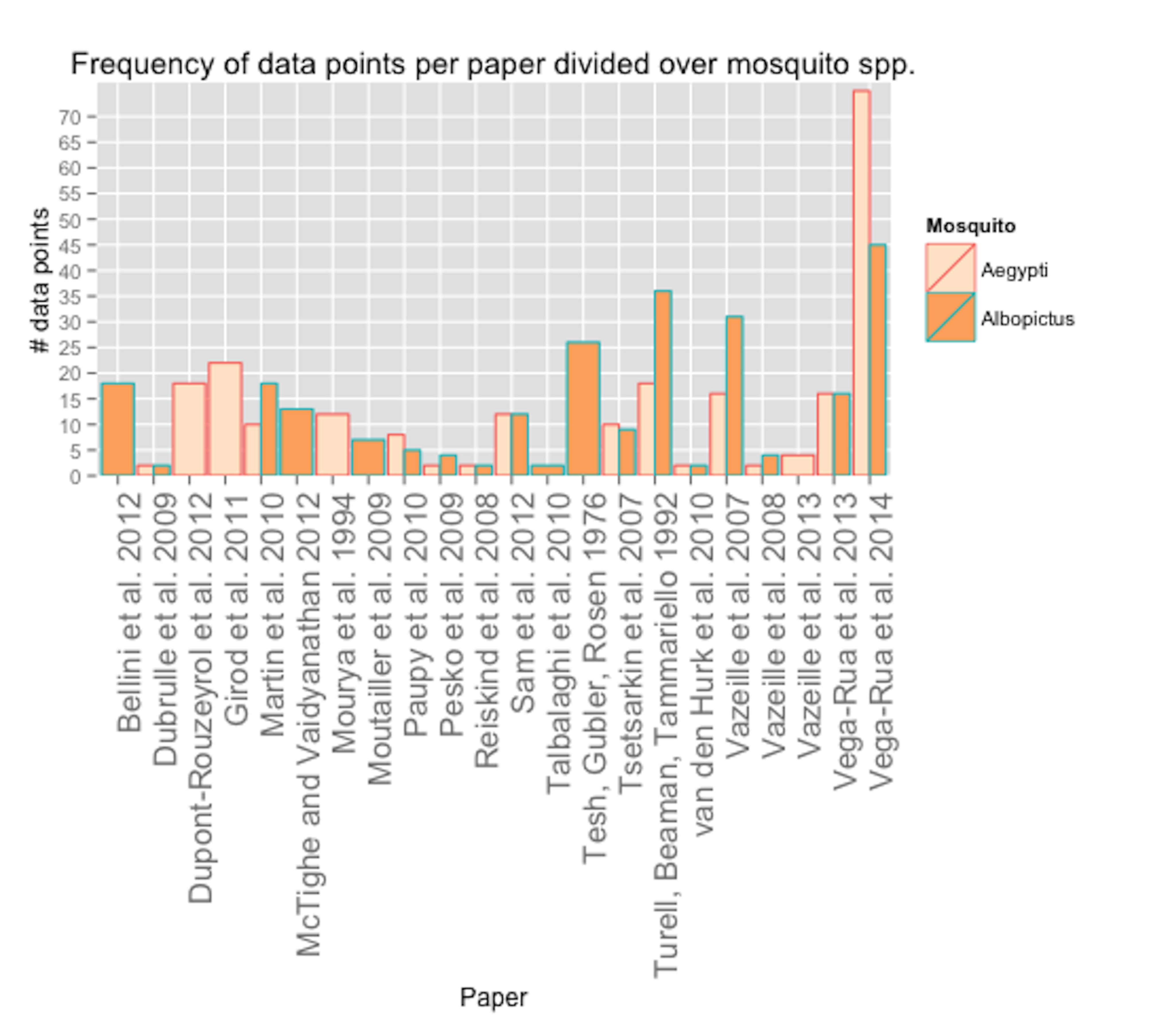

Supplement: Figure S1 — The number of vector competence data points (y-axis) for each study (x-axis). The papers are subdivided (colored bars) depending on whether the data was for Ae. aegypti or Ae. albopictus or both. (TIFF) [file pone.0110538.s001.tiff]

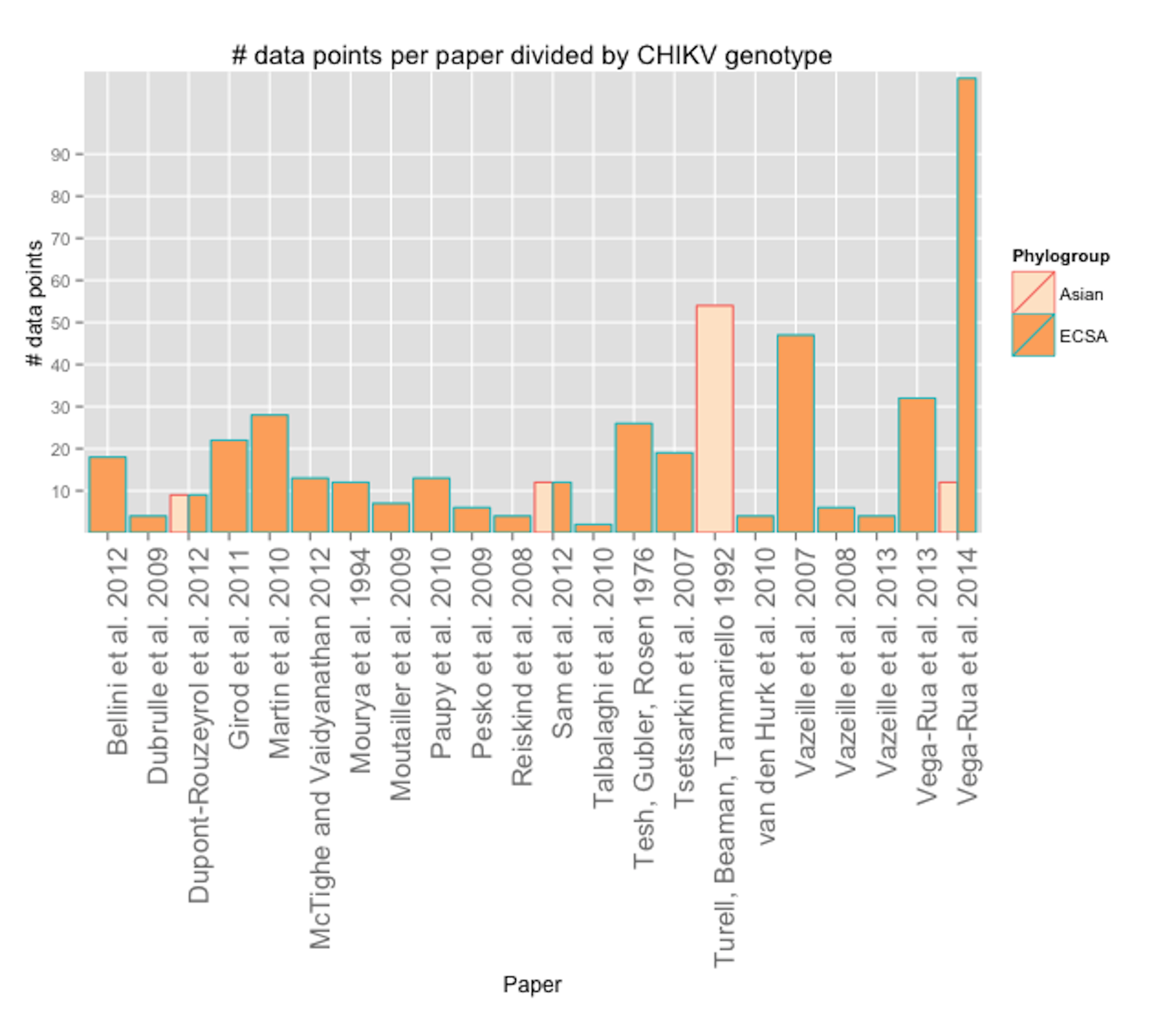

Supplement: Figure S2 — The number of vector competence data points (y-axis) for each study (x-axis). Studies are divided (colored bars) depending on whether the data is for the Asian or ECSA genotype. (TIFF) [file pone.0110538.s002.tiff]

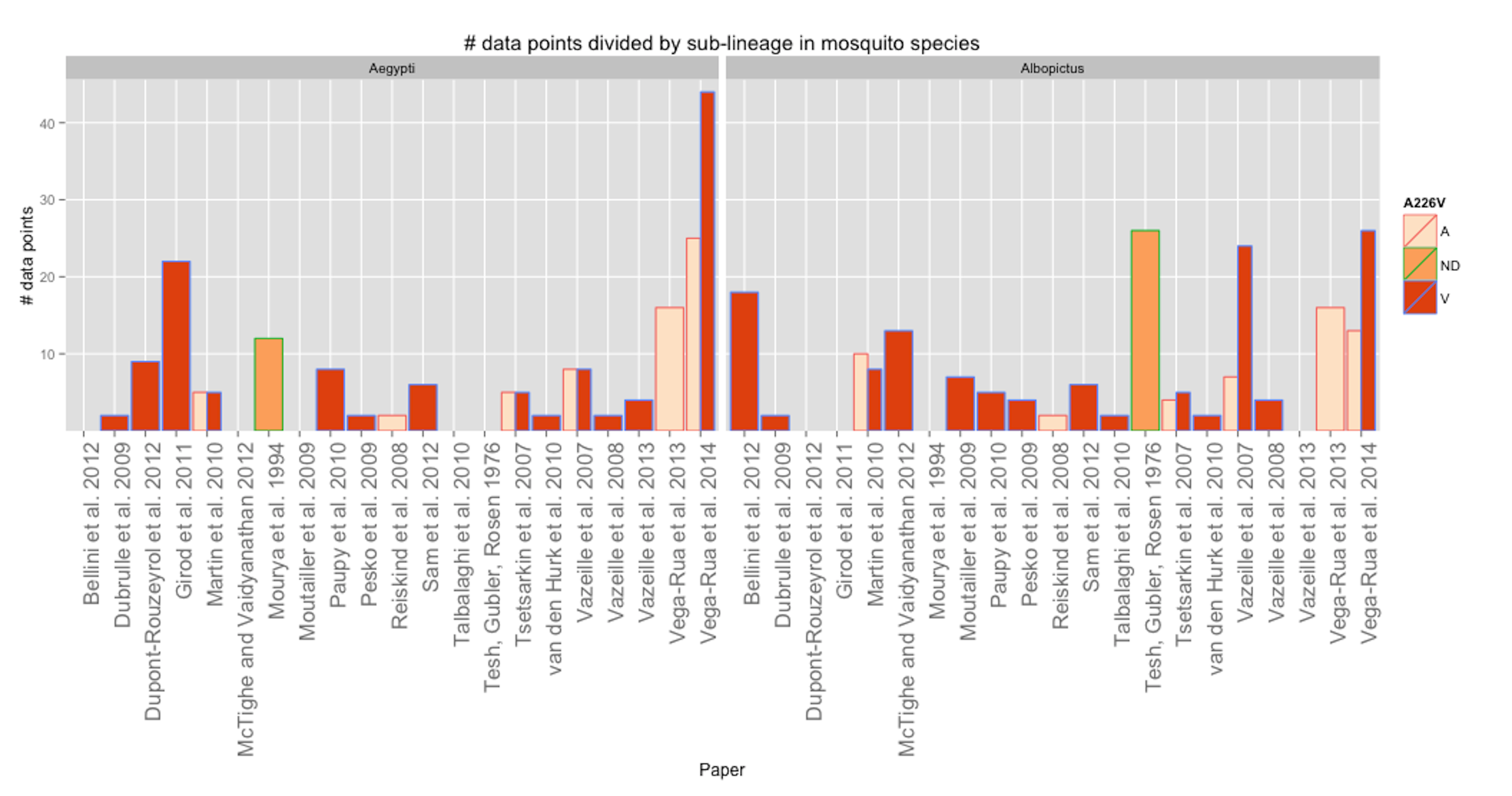

Supplement: Figure S3 — The number of vector competence data points (y-axis) for each study (x-axis). The studies are divided (colored bars) based on whether the data corresponds to the ECSA-A (A), ECSA-V (V) sublineage of the ECSA genotype or if the dilineation in the ECSA genotype was not determined (ND). (TIFF) [file pone.0110538.s003.tiff]

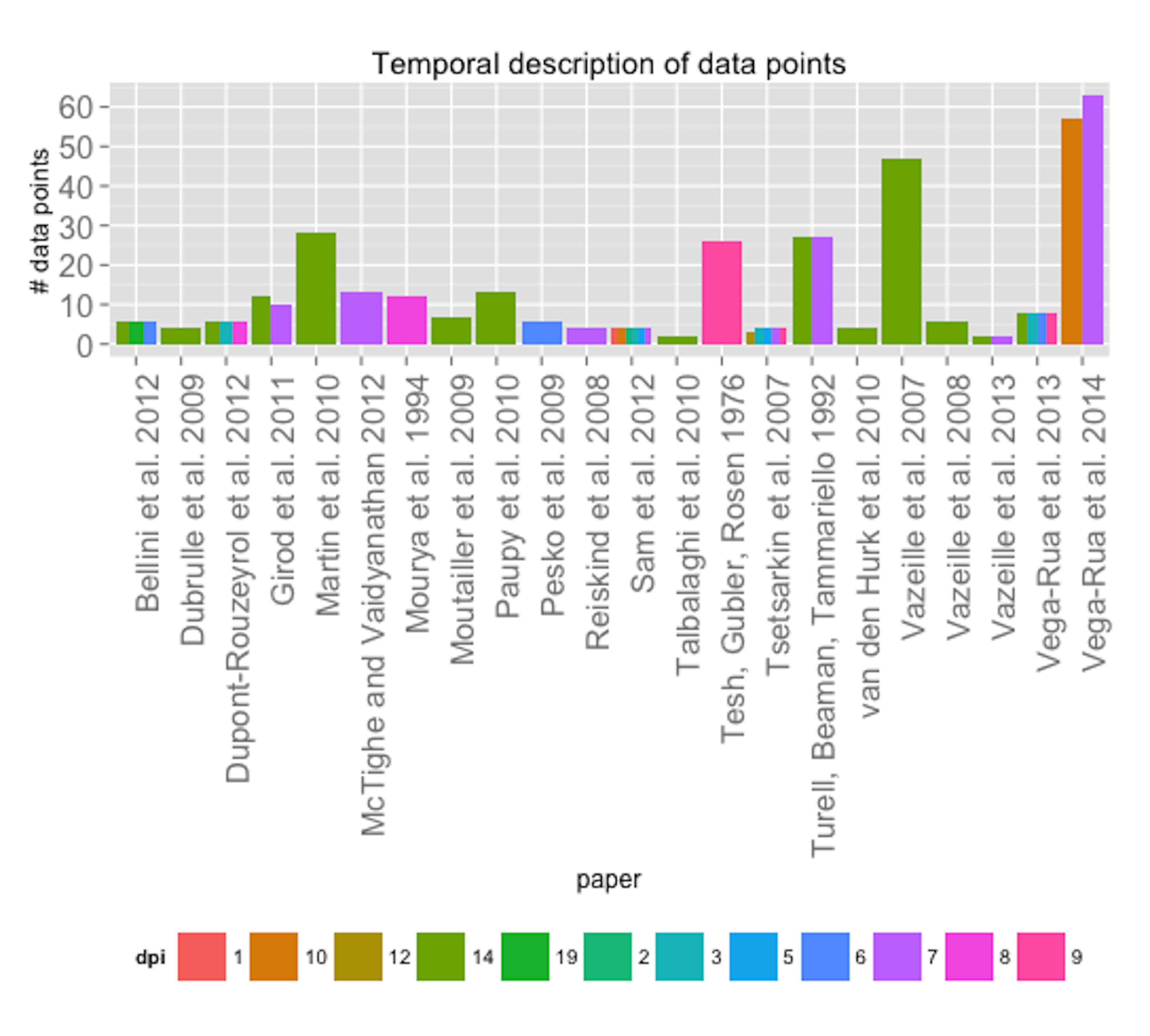

Supplement: Figure S4 — The number of vector competence data points (y-axis) for each study (x-axis) divided (colored bars) by the day on which the data point was assessed. (TIFF) [file pone.0110538.s004.tiff]

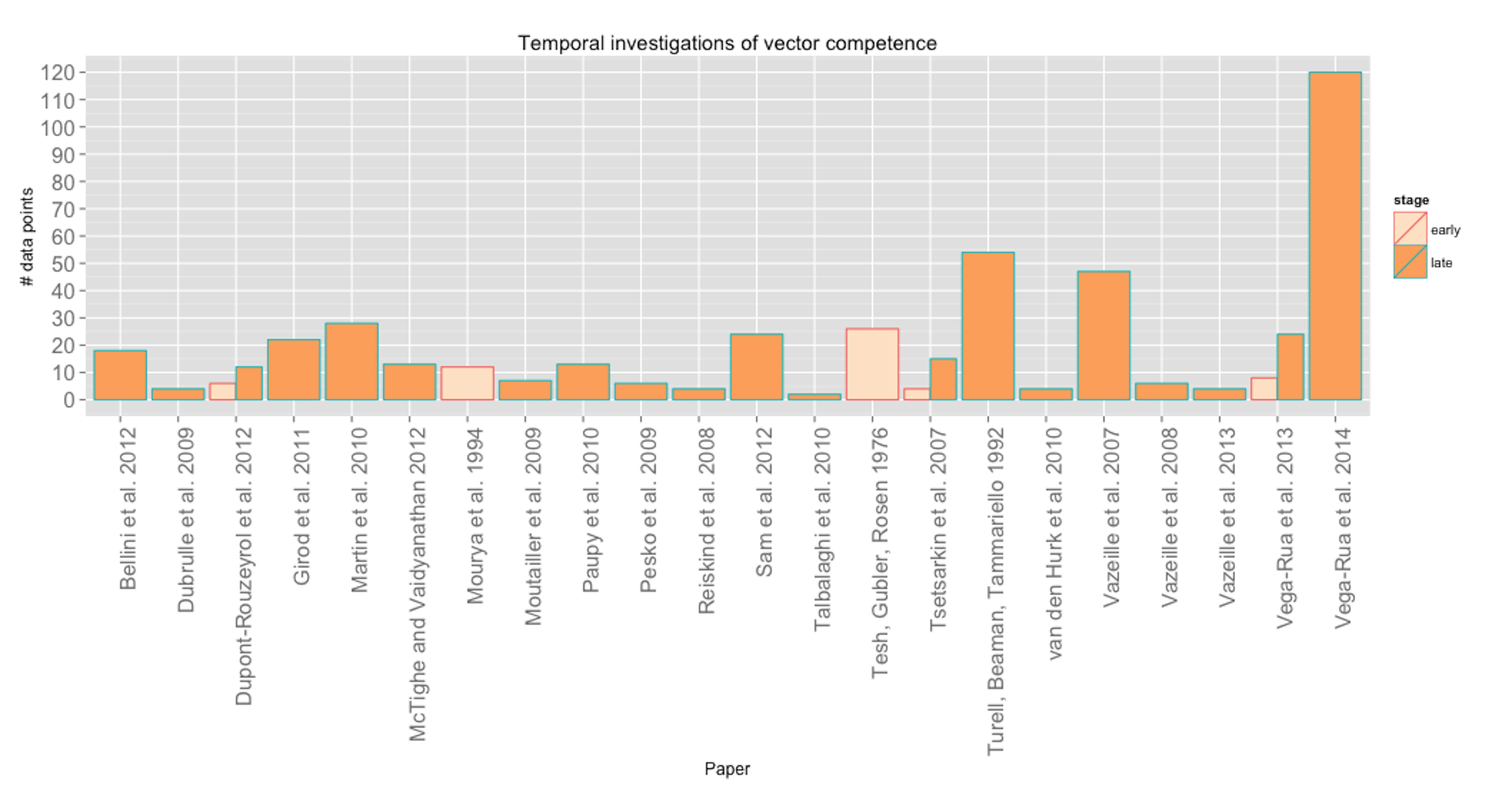

Supplement: Figure S5 — The number of vector competence data points (y-axis) for each study (x-axis) divided (colored bars) depending on whether the dissemination determination was done during the early stage of infection (≤7 days post exposure) or late stage (>7 days). (TIFF) [file pone.0110538.s005.tiff]

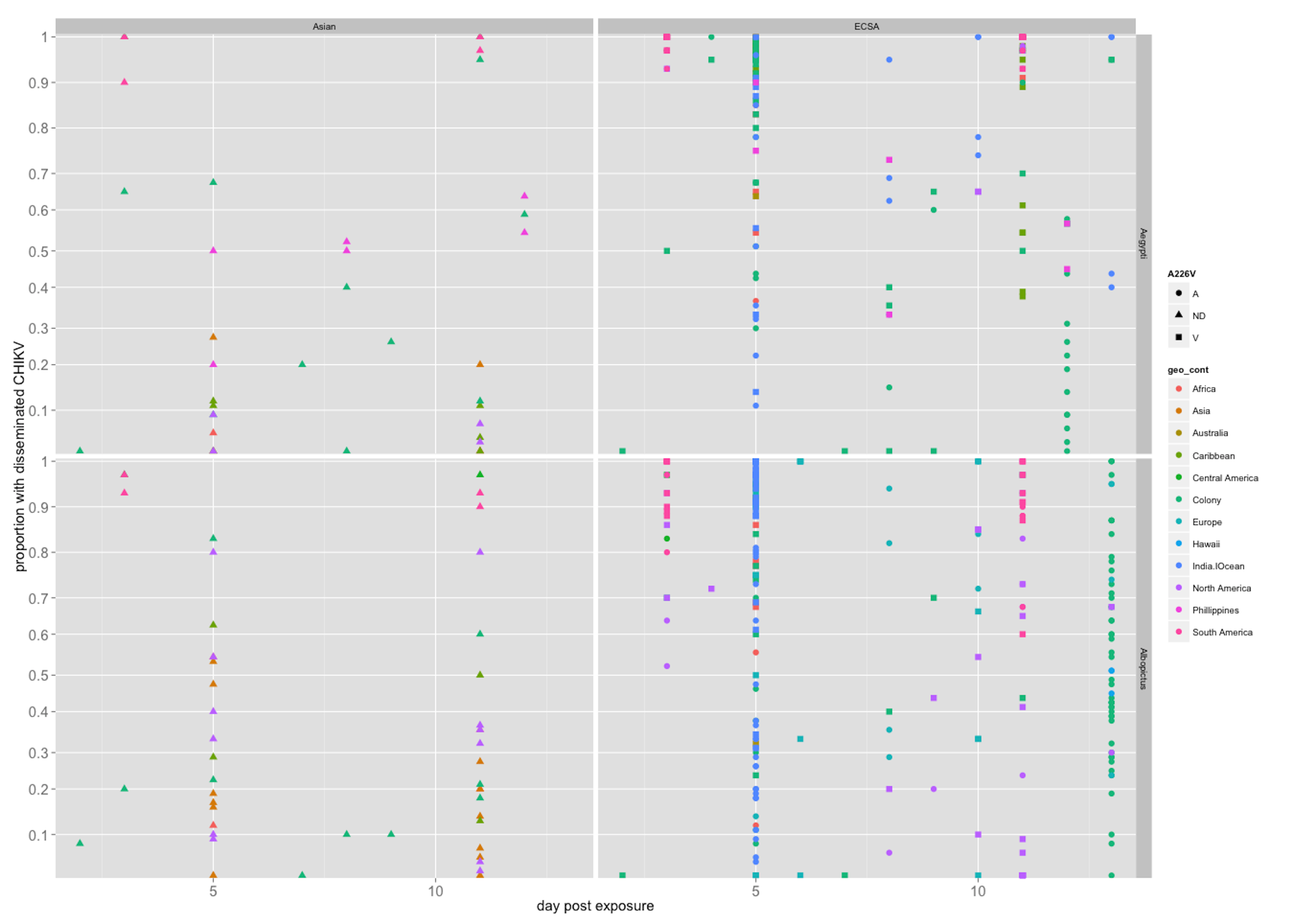

Supplement: Figure S6 — Scatterplot of data points when subdivided by CHIKV genotype (columns) and mosquito species (rows). Color denotes the continental or regional origin of the mosquito strains utilized (or denoted as ‘colony’ if applicable) and shape of the point denotes sub-lineage of ECSA (A or V) or ND (not-determined) if Asian genotype. (TIFF) [file pone.0110538.s006.tiff]
